# Synthesis of Reactive Water-Soluble Narrow-Band-Gap Polymers for Post-Crosslinking

**DOI:** 10.3390/polym12020313

**Published:** 2020-02-03

**Authors:** Hao-xuan Guo, Takehiro Ohashi, Yusuke Imai, Hiroyuki Aota

**Affiliations:** Department of Chemistry and Materials Engineering, Kansai University, Suita, Osaka 564-8680, Japan; miwanight25@gmail.com (T.O.); imaiyusuke.425752@gmail.com (Y.I.); aota@kansai-u.ac.jp (H.A.)

**Keywords:** π-conjugated polymer, narrow-band-gap polymer, post-crosslinking

## Abstract

In this study, water-soluble, narrow-band-gap polymers containing reactive groups were prepared by the addition-condensation of pyrrole (Pyr), benzaldehyde-2-sulfonic acid sodium salt (BS), and terephthalaldehydic acid (TPA) or *p*-hydroxybenzaldehyde (*p*-HB). TPA and *p*-HB were used for the post-crosslinking reaction between polymers. The polymers were characterized by employing various analyses such as ^1^H-NMR, thermal gravimetric analysis (TGA), and UV-Vis-NIR. The *E*_g_ values of polymers estimated from the absorption edges were 0.55 and 0.62 eV. The post-crosslinking reaction is important for preventing resolubilization and for developing an electron conducting route between the polymer chains. Herein, the post-crosslinked polymer was observed to maintain its narrow-band-gap and conductivity was increased 46 times compared to that observed before crosslinking.

## 1. Introduction

Conductive polymers are important fundamental materials that facilitate the development of modern organic electronics. The Nobel Prize in Chemistry in 2000 was shared by Alan J. Heeger, Alan G. MacDiarmid, and H. Shirakawa who were the first to develop conductive polymers. These materials are now being researched and developed worldwide [1]. Currently, conductive polymers are being used in various applications including solar cells [2,3,4], organic EL [5], sensors [6,7], and solid electrolyte capacitors [8,9].

Characteristic π-conjugated polymers including polythiophene, polypyrrole, and polyacetylene doped with oxidizing or reducing agents, generate charge carriers and consequently become conductive polymers [1]. A doping level is generated between the valence and conduction bands upon doping. By decreasing the band gap energy, charge carriers such as holes and electrons can easily move between different energy levels. If the band gap is sufficiently narrow, high electrical conductivity can be achieved even when undoped. Narrow-band-gap polymers have been studied for realization of an intrinsic conducting polymer [10,11,12,13,14,15,16,17,18]. In our previous studies, the synthesis of water-soluble, narrow-band-gap polymers with band-gap values of ˂0.19 eV was developed [19]. In addition, the band-gap values of these polymers were continuously controlled from 0.3 to 1.1 eV in aqueous solution [20]. However, the conductivity of the narrowest-band-gap polymer was insignificant due to the negligible amount of thermally excited conductive electrons and a lack of electron conducting routes between the polymer chains.

PEDOT [poly (3,4-ethylenedioxythiophene) doped with poly (4-styrenesulfonic acid) (PSS)] is one of the most successful conductive polymers [21]. It has been widely used in solar cells [2,3,4], solid electrolyte capacitors [8,9], and hole injection layers of organic EL [5]. However, PEDOT/PSS exhibits poor solubility in organic solvents, forming a colloidal dispersion [22]; Consequently, it is difficult to form a thin film and perform impregnation of etched pits when preparing an aluminum solid electrolytic capacitor (smaller than colloidal particles) [23]. Herein, we focused on narrow-band-gap polymers synthesized by the addition-condensation of pyrrole and benzaldehyde-2-sulfonic acid sodium salt, which are soluble in water and some organic solvents. When preparing electronic devices using polymers, solubility control remains important. Cast and spin-coating methods are widely used to prepare devices with thin films of soluble polymers. However, the stability of the thin films in the devices using electrolyte solutions are poor due to resolubilization. Post-crosslinking is important for preventing resolubilization and preparing electron conducting routes between the polymer chains [24,25]. Herein, we demonstrate the synthesis of reactive water-soluble narrow-band-gap polymers for post-crosslinking. The post-crosslinked polymer was observed to maintain a narrow-band-gap and its conductivity increased compared to the value measured before crosslinking. 

## 2. Materials and Methods 

### 2.1. Materials 

Unless stated otherwise, reagents and solvents were purchased and used without purification. Benzaldehyde-2-sulfonic acid sodium salt (BS) and terephthalaldehydic acid (TPA) were purchased from Tokyo Chemical Industry (Tokyo, Japan). *p*-toluenesulfonic acid monohydrate (*p*-TS) and *p*-hydroxybenzaldehyde (*p*-HB) were purchased from FUJIFILM Wako Pure Chemical Corporation (Osaka, Japan). Pyrrole (Pyr) was purchased from Sigma-Aldrich Japan (Tokyo, Japan). Pyr monomer was purified by distillation.

### 2.2. Analytical Method

UV-Vis-NIR spectra were measured using a V-670 spectrophotometer (JASCO, Tokyo, Japan) and the ^1^H-NMR spectra were recorded using a JEOL ECZ-400 spectrometer (Tokyo, Japan). The TGA spectrum was measured using a differential scanning calorimeter DSC6000 (HITACHI, Tokyo, Japan). Before measuring TGA, the polymers were dried under vacuum overnight. 

The reduced viscosity (η_sp_/C) was measured using an Ubbelohde-type viscometer with a 4.0 g/L sample solution at 30 °C. The molecular weights of the polymers were roughly estimated using the Equation (1)

η_sp_/C = kMw^α^ (1)

where, k (1.16 × 10^−5^) and α (0.894) were approximated from the η_sp_/C and *M*_w_ values determined via ultracentrifugal analysis. 

**Figure 1:**
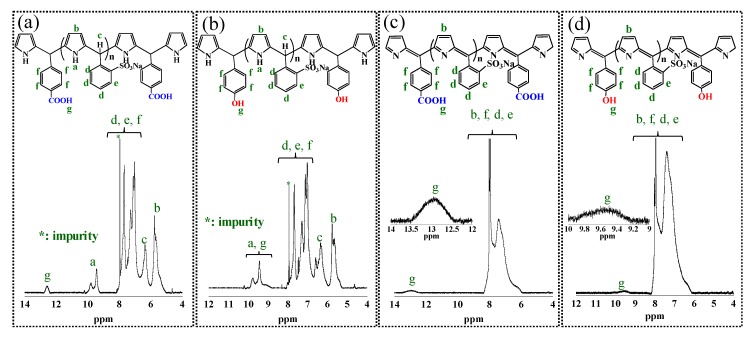
^1^H-NMR spectra of polymers in DMSO-d_6_.

To prepare the pressed samples (Figure 1), the polymer (0.010 g) was filled into ClearDisc CD-10 and pressurized to 10 kN using a hydraulic press. The electric resistance of samples was determined using a four-probe resistivity measurement device (MCP-T410, Mitsubishi Chemical Holdings, Tokyo, Japan). The conductivity was calculated by Equations (2) and (3)

*ρ*_V_ = R × RCF × t (2)

σ = 1/*ρ*_V_ (3)

where, *ρ*_V_: volume resistivity (Ωcm), R: resistance (Ω), RCF: resistivity correction factor, *t*: thickness (cm), σ: conductivity (S/cm)

Band gap was estimated from the absorption edge using the Equation (4)

(αhv)^2^ = (const)(hv − *E*_g_) (4)

where, α, v, and *E*_g_ are the absorption coefficient, light frequency, and band gap energy, respectively. 

### 2.3. Synthesis

#### 2.3.1. Narrow-Band-Gap Polymers Containing Reactive Groups 

**P4** was prepared as follows: a solution containing *p*-toluenesulfonic acid monohydrate (0.095 g, 0.50 mmol) dissolved in DMF (1.0 mL), which was added to another solution containing pyrrole (Pyr, 0.335 g, 5.0 mmol) and benzaldehyde-2-sulfonic acid sodium salt (BS, 1.25 g, 6.0 mmol) dissolved in DMF (5.0 mL) at 10 °C. The mixed solution was stored in the dark at 10 °C for 24 h. After 24 h, a solution containing terephthalaldehydic acid (TPA, 0.225 g, 1.80 mmol) and pyrrole (Pyr, 0.101 g, 1.5 mmol) dissolved in DMF (1.0 mL) was added to reaction mixture and stored in the dark for 6 h. Isopropyl alcohol (80 mL) was then added to the reaction mixture. The resulting precipitate, a non-conjugated polymer (**P2**), was purified by two reprecipitations from DMF/isopropyl alcohol (8 mL/80 mL) and two precipitations from water/isopropyl alcohol (6 mL/80 mL), and subsequently dissolved in water. The polymer (**P2**) was obtained by freeze-drying (1.395 g, *M*_w_ = 13000). **P3** (1.237 g, *M*_w_ = 8000) was prepared in a similar manner. A solution of chloranil (1.171 g, 4.7 mmol) dissolved in DMF (21 mL) was then added to a solution of the non-conjugated polymer (**P2**, 1.225 g) dissolved in DMF (3.0 mL). The reaction mixture was stirred at 30 °C for 6 h and poured into toluene (80 mL). The resulting precipitate was purified by reprecipitation from DMF/toluene (8 mL/80 mL) four times, DMF/isopropyl alcohol (8 mL/80 mL), and water/isopropyl alcohol (6 mL/80 mL). The π-conjugated polymer (**P4**) was obtained by freeze-drying (0.27 g, *M*_w_ = 36000) and the conductivity of the pressed **P4** was 2.3 × 10^−7^ S/cm. **P5** (0.31 g, *M*_w_ = 20000) was prepared in a similar manner and the conductivity of the pressed **P5** was 1.8 × 10^−7^ S/cm.

#### 2.3.2. Post-Crosslinking Reaction of the Narrow-Band-Gap Polymers 

**P6** was prepared by adding *p*-toluenesulfonic acid monohydrate (0.05 g, 0.02 mmol) to a solution containing **P4** (0.10 g) and **P5** (0.10 g) dissolved in DMF (super dehydrated, 3 mL). The mixed solution was subsequently stirred at 100 °C for 24 h and the insoluble part was separated by suction filtration and washed with water and methanol. The post-crosslinking polymer (**P6**) was obtained by vacuum drying (0.042 g). The gel-fraction was 20%, and the conductivity of the pressed **P6** was 9.0 × 10^−6^ S/cm. 

## 3. Results and Discussion

**Scheme 1:**
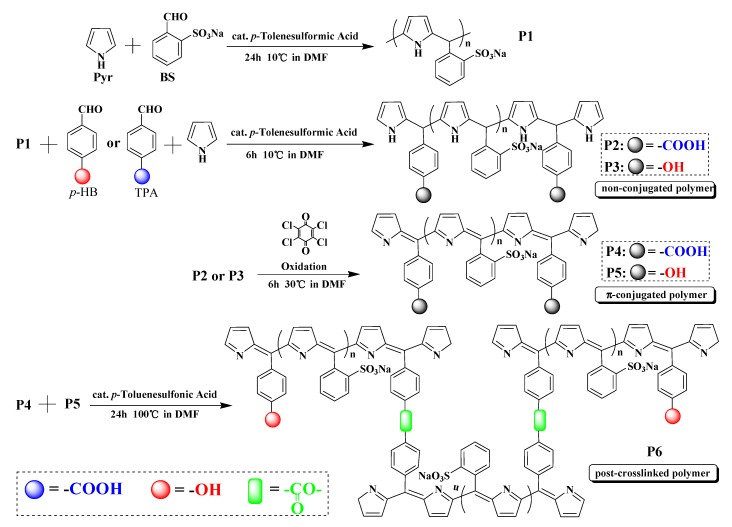
Synthetic routes of narrow-band-gap polymers.

Water-soluble, narrow-band-gap polymers (**P4** and **P5**) containing reactive groups were prepared by the addition-condensation of Pyr, BS, and TPA or *p*-HB. TPA and *p*-HB were used for the post-crosslinking reaction between polymers. The synthetic routes of the polymers are shown in Scheme 1 and the details are further described in the Section 2.3.1. 

Figure 1 and Figure S1 (in supplementary materials) show the ^1^H-NMR spectra of the prepared polymers (**P1**–**P5**). For **P2** (Figure 1a), the peaks at 9–10 and 5.5 ppm were assigned to the Pyr protons (a and b) and the peaks at 13 ppm were assigned to the protons in the carboxy group (g). However, the signals of BS protons (d and e) and TPA protons (f) in the polymer were not visible due to spectral broadening and overlapping peaks. For **P3** (Figure 1b), the peak at 5.5 ppm was assigned to the Pyr protons (b). The BS (d and e), *p*-HB (f), and *p*-HB protons (g: hydroxy) in the polymer were not assigned due to spectral broadening and overlapping peaks. After oxidation reaction, the peak at 13 ppm was assigned to the proton in the carboxy group (g) (Figure 1c), the peak at 9.5 ppm was assigned to the proton in the hydroxy group (g) (Figure 1d), and the other peaks were consistent with the conjugated polymer Pyr (non) (Figure S2 in supplementary materials). Therefore, **P4** and **P5** were identified, and the oxidation reaction of the polymers did not affect overall functionality. In addition, peak broadening was observed from 6 to 8.2 ppm due to the oxidation of the π-conjugated polymers, which caused toughening of the main chain due to slowed molecular motion. 

**Figure 2:**
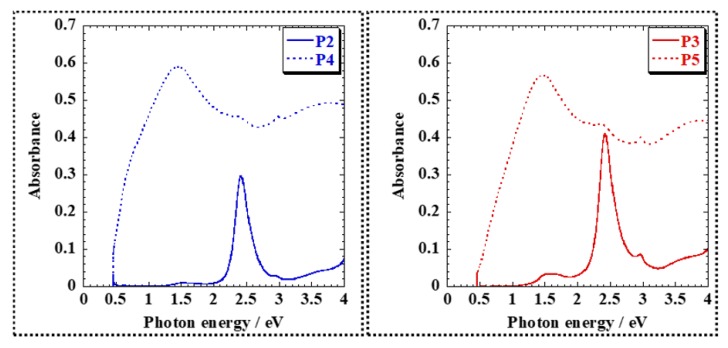
UV-Vis-NIR spectra of polymers (**P2**-**P5**) dissolved in phosphate buffer. [polymer] = 4.0 g/L, cell length: 0.1 mm.

Figure 2 and Figure S3 (in supplementary materials) show the absorption spectra of **P4** and **P5** dissolved in a phosphate buffer (25 mM KH_2_PO_4_ and 25 mM Na_2_HPO_4_; pH 6.9) to avoid self-acid doping. The absorption profiles of the polymers in Figure 2 and Figure S3 were very broad. We showed that the longer-wavelength absorptions were due to π–π * excitation of the expanded π-conjugation. The *E*_g_ values estimated from the absorption edges shown in Figure 2 and Figure S4 (in supplementary materials) were 0.55 and 0.62 eV, respectively. In other words, the synthesized conjugated polymers were narrow band gap polymers.

**Figure 3:**
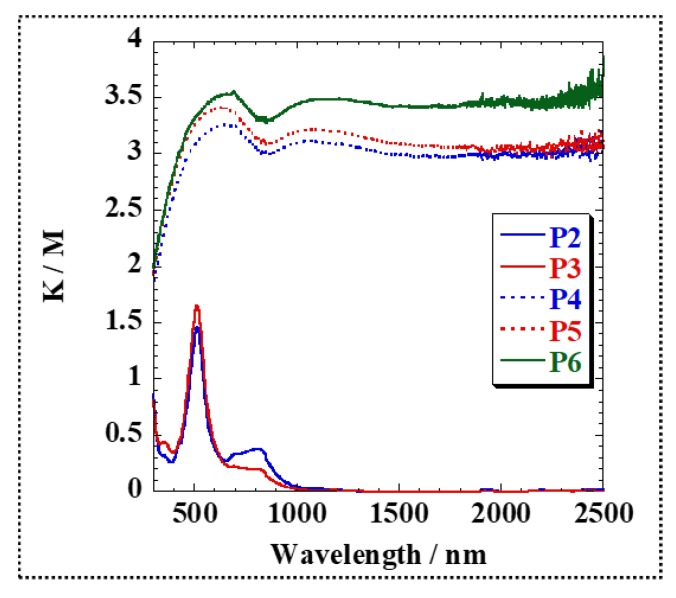
UV-Vis-NIR spectra of the powdered polymers, transformed using diffuse reflection spectra.

The post-crosslinking reaction of **P4** and **P5** was performed (Scheme 1). The details are further described in the Section 2.3.2. **P6** was prepared by post-crosslinking reaction of the water-soluble polymers (**P4** and **P5**) and was insoluble in water and almost all organic solvents (DMF, methanol, ethanol, acetone, hexane, and chloroform). Resolubility was prevented by post-crosslinking and because **P6** was insoluble, we measured the diffuse reflection of the polymer powder using an integrating sphere (Figure S6 in supplementary materials). The UV-Vis-NIR spectra (Figure 3) of the powdered polymers were transformed using the corresponding diffuse reflection spectra (Figure S5 in supplementary materials). Compared with **P2** and **P3** (the nonconjugated polymer), the absorption profiles of **P6** were very broad and similar to those of **P4** and **P5**. In other words, the post-crosslinked polymer **P6** maintained its narrow-band-gap. In addition, we examined the thermal decomposition of **P6** using TGA, **P6** showed higher heat resistance than those of **P4** and **P5** (Figure S6 in supplementary materials). Finally, the conductivity of the pressed polymer samples was compared. The conductivity of **P6** (9.0 × 10^−6^ S/cm) was 46 times higher than that of the polymer before crosslinking **P4** + **P5** (1.9 × 10^−7^ S/cm). The reason for the improved conductivity is due to the electron conducting routes between the polymer chains being improved via post-crosslinking. 

## 4. Conclusions

In conclusion, we succeed in preparing a reactive (post crosslinkable) water-soluble narrow-band-gap polymer via addition-condensation of Pyr, BS, and TPA or *p*-HB. The post-crosslinked polymer exhibited a high heat resistance, insolubility, and was able to maintain its narrow-band-gap. The conductivity of the post-crosslinked polymer increased by 46 times, as compared to that observed before crosslinking. In the future, we plan to improve the conductivity of these polymers for utilization in various applications of materials science, especially in solid electrolyte capacitors and solar cells.

## Supplementary Materials

The following are available online at https://www.mdpi.com/2073-4360/12/2/313/s1, Figure S1: ^1^H-NMR spectrum of **P1** in DMSO-d_6_; Figure S2: ^1^H-NMR spectrum of Pyr(non) in DMSO-d_6_; Figure S3: UV-Vis-NIR spectra of polymers (**P2**-**P5**) dissolved in phosphate buffer, [polymer] = 4.0 g/L, cell length: 0.1 mm; Figure S4: (hvα)^2^ versus photon energy around the absorption edge of polymers **(P4**, **P5**) dissolved in phosphate buffer, [polymer] = 4.0 g/L, cell length: 0.1 mm; Figure S5: Diffuse reflection spectra of the powdered polymers; Figure S6: TGA curves of **P4-6** with a heating rate of 5 °C/min in air.

## References

[1] MacDiarmid A.G. (2001). Synthetic Metals: A Novel Role for Organic Polymers (Nobel Lecture). Angew. Chem. Int. Ed..

[2] Cheng Y.-J., Yang S.-H., Hsu C.-S. (2009). Synthesis of conjugated polymers for organic solar cell applications. Chem. Rev..

[3] Li G., Zhu R., Yang Y. (2012). Polymer solar cells. Nat. Photonics.

[4] Wu Z., Sun C., Dong S., Jiang X.-F., Wu S., Wu H., Yip H.-L., Huang F., Cao Y. (2016). n-Type water/alcohol-soluble naphthalene diimide-based conjugated polymers for high-performance polymer solar cells. J. Am. Chem. Soc..

[5] Burroughes J.H., Bradley D.D.C., Brown A.R., Marks R.N., Mackay K., Friend R.H., Burn P.L., Holmes A.B. (1990). Light-emitting diodes based on conjugated polymers. Nature.

[6] Shoji E., Freund M. (2002). Potentiometric Saccharide Detection Based on the pKa Changes of Poly (aniline boronic acid). J. Am. Chem. Soc..

[7] Luo S., Ali E.M., Tansil N.C., Yu H., Gao S., Kantchev E.A.B., Ying J.Y. (2008). Poly(3,4-Ethylenedioxythiophene) (PEDOT) Nanobiointerfaces: Thin, Ultrasmooth, and Functionalized PEDOT Films with in Vitro and in Vivo Biocompatibility. Langmuir.

[8] Kudoh Y., Akami K., Matsuya Y. (1999). Solid electrolytic capacitor with highly stable conducting polymer as a counter electrode. Synth. Met..

[9] Wakabayashi T., Katsunuma M., Kudo K., Okuzaki H. (2018). pH-Tunable High-Performance PEDOT: PSS Aluminum Solid Electrolytic Capacitors. ACS Appl. Energy Mater..

[10] Kobayashi M., Colaneri N., Boysel M., Wudl F., Heeger A.J. (1985). The electronic and electrochemical properties of poly(isothianaphthene). J. Chem. Phys..

[11] Becker R., Blöchl G., Bräunling H. (1990). Polyheteroarylmethines, Syntheses and Physical Properties. NATO ASI Ser. E.

[12] Bräunling H., Becker R., Blöchl G. (1991). Polyarylmethines; Synthesis and Physical Properties. Synth. Met..

[13] Meyers F., Adant C., Toussaint J.M., Bredas J.L. (1991). AB initio study of the structural, electronic, and nonlinear optical properties of pyrrole derivatives. Synth. Met..

[14] Toussaint J.M., Brédas J.L. (1993). Theoretical analysis of the geometric and electronic structure of small-band-gap polythiophenes: poly (5,5’-bithiophene methine) and its derivatives. Macromolecules.

[15] Tanaka S., Yamashita Y. (1995). Syntheses of narrow band gap heterocyclic copolymers of aromatic-donor and quinonoid-acceptor units. Synth. Met..

[16] Akoudad S., Roncali J. (1998). Electrogenerated poly(thiophenes) with extremely narrow bandgap and high stability under n-doping cycling. Chem. Commun..

[17] Hong S.Y. (2000). Zero Band-Gap Polymers:  Quantum-Chemical Study of Electronic Structures of Degenerate π-Conjugated Systems. Chem. Mater..

[18] Casado J., Ortiz R.P., Delgado M.C.R., Hernández V., Navarrete J.T.L., Raimundo J.-M., Blanchard P., Allain M., Roncali J. (2005). Alternated Quinoid/Aromatic Units in Terthiophenes Building Blocks for Electroactive Narrow Band Gap Polymers. Extended Spectroscopic, Solid State, Electrochemical, and Theoretical Study. J. Phys. Chem. B.

[19] Aota H., Ishikawa T., Amiuchi Y., Yano H., Kunimoto T., Matsumoto A. (2010). Band Gap and Absorption Profile Change by Changing Molecular Weight and Conformation of Water-soluble Narrow-band-gap Polymers. Chem. Lett..

[20] Aota H., Ishikawa T., Maki Y., Takaya D., Ejiri H., Amiuchi Y., Yano H., Kunimoto T., Matsumoto A. (2011). Continuous Band Gap Control from 0.3 to 1.1 eV of π-Conjugated Polymers in Aqueous Solution. Chem. Lett..

[21] Elschner A., Kirchmeyer S., Lovenich W., Merker U., Reuter K. (2010). PEDOT: Principles and Applications of an Intrinsically Conductive Polymer.

[22] Takano T., Masunage H., Fujiwara A., Okuzaki H., Sasaki T. (2012). PEDOT Nanocrystal in Highly Conductive PEDOT: PSS Polymer Films. Macromolecules.

[23] Yan H., Arima S., Mori Y., Kagata T., Sato H., Okuzaki H. (2009). Poly(3,4-ethylenedioxythiophene)/poly(4-styrenesulfonate): Correlation between colloidal particles and thin films. Thin Solid Films.

[24] Rumer J.W., McCulloch I. (2015). Organic photovoltaics: Crosslinking for optimal morphology and stability. Materials Today.

[25] Lee H.-J., Jo Y.-R., Kumar S., Yoo S.J., Kim J.-G., Kim Y.-J., Kim B.-J., Lee J.-S. (2016). Close-Packed Polymer Crystals from Two-Monomer-Connected Precursors. Nat. Commun..

