# Supplementary material for: Synthesis of Reactive Water-Soluble Narrow-Band-Gap Polymers for Post-Crosslinking
Source: Polymers (Basel). 2020 Feb 3;12(2):313. doi: 10.3390/polym12020313 (PMC7077490; doi:10.3390/polym12020313)
Supplement: Supplementary file 1 [file polymers-12-00313-s001.pdf]

Supplementary Materials

Synthesis of Reactive Water-soluble Narrow-Band-Gap Polymers for Post-crosslinking

Hao-xuan Guo,[*1] Takehiro Ohashi,[1] Yusuke Imai,[1] and Hiroyuki Aota[*1]

[1]*Department of Chemistry and Materials Engineering,*

Kansai University, Suita, Osaka 564-8680, Japan

Correspondence: hx-guo@kansai-u.ac.jp

(1) ^{1}H-NMR spectra of the non-conjugated polymers

P1: **Figure S1** shows the chemical structure and ^{1}H-NMR spectrum of **P1**. The peaks at 9–10, 6.5, and 5.5 ppm were assigned to the Pyr protons (a, c, and b). The peaks at 7.08 and 7.73 ppm were assigned to the BS protons (d and e).

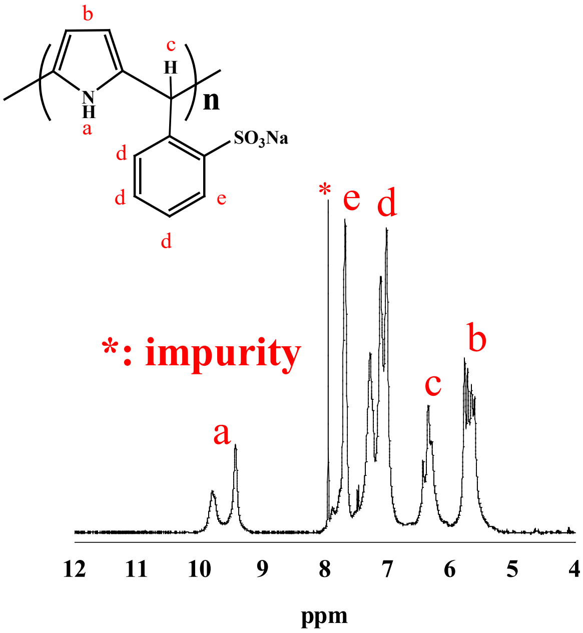

Figure S1. ^{1}H-NMR spectrum of **P1** in DMSO-d$_6$.

(2) ^{1}H-NMR spectrum of the narrow-band-gap polymer

Figure S2 shows the chemical structure and ^{1}H-NMR spectrum of the π-conjugated polymer **Pyr(non)**. Spectral broadening from 6 to 8.2 ppm was observed for **Pyr(non)**. However, no characteristic N-H peaks at approximately 9.5 ppm in the ^{1}H-NMR spectrum were observed because the polymer became a π-conjugated polymer upon oxidation.

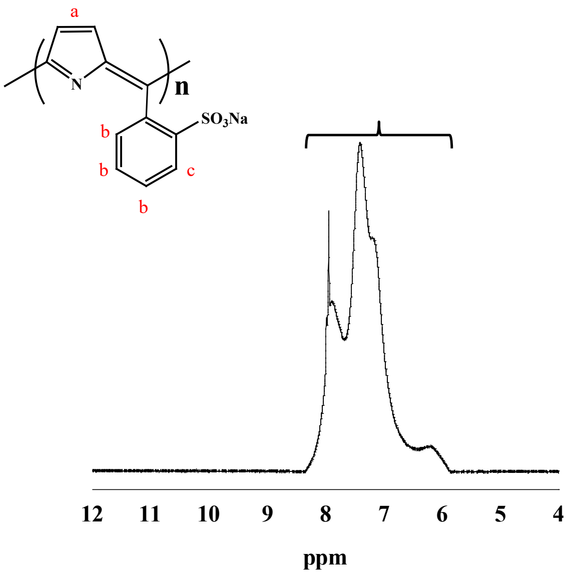

Figure S2. ^{1}H-NMR spectrum of **Pyr(non)** in DMSO-d_6.

(3) UV-Vis-NIR spectra of P2-P5

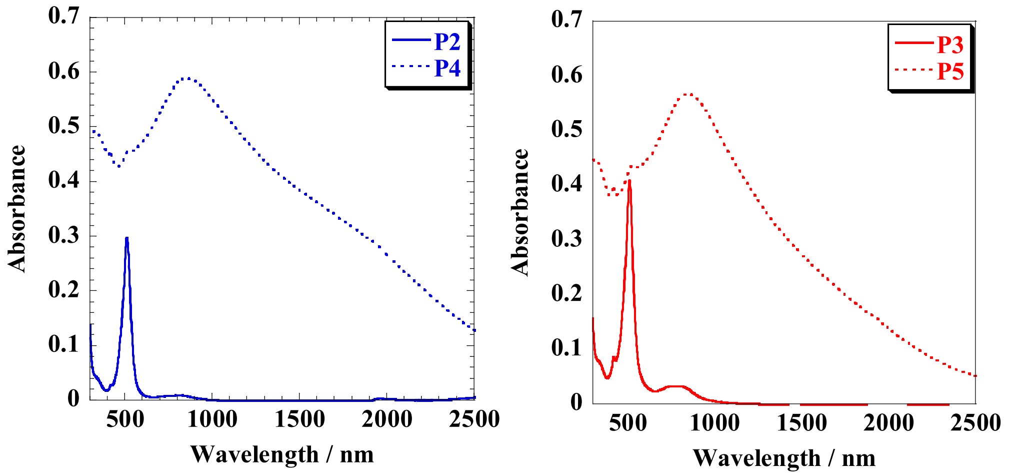

Figure S3. UV-Vis-NIR spectra of polymers (**P2-P5**) dissolved in phosphate buffer, [polymer] = 4.0 g/ L, cell length: 0.1 mm.

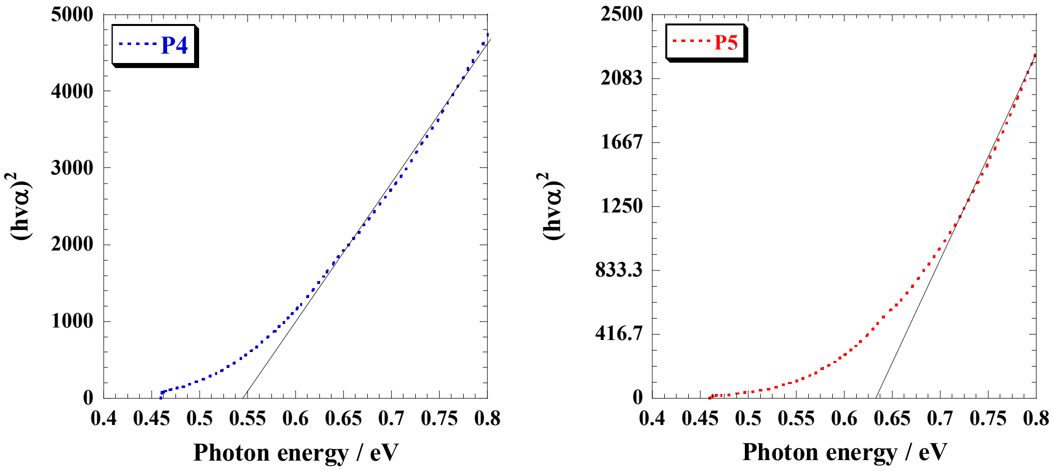

Figure S4. $(h\nu\alpha)^2$ versus photon energy around the absorption edge of polymers (**P4**, **P5**) dissolved in phosphate buffer, [polymer] = 4.0 g/ L, cell length: 0.1 mm.

(4) Diffuse reflection spectra of P2, P3, P4, P5, and P6

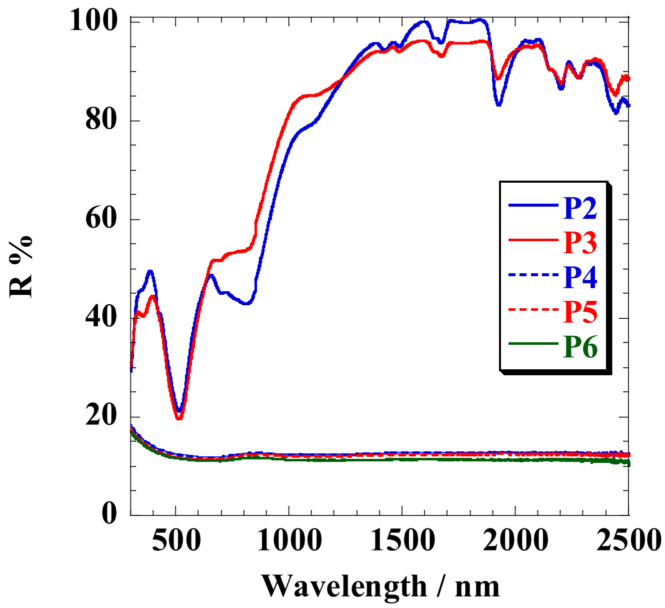

For the powdered samples, reflection spectra could not be measured. We measured the diffuse reflectance spectra using an integrating sphere. The vertical axis was converted from reflectance to absorption using the Kubelka-Munk conversion (KM conversion).
Before measuring the diffuse reflection, the polymer powders were dried under vacuum overnight.

Figure S5. Diffuse reflection spectra of the powdered polymers.

(5) TGA curves of P4-P6

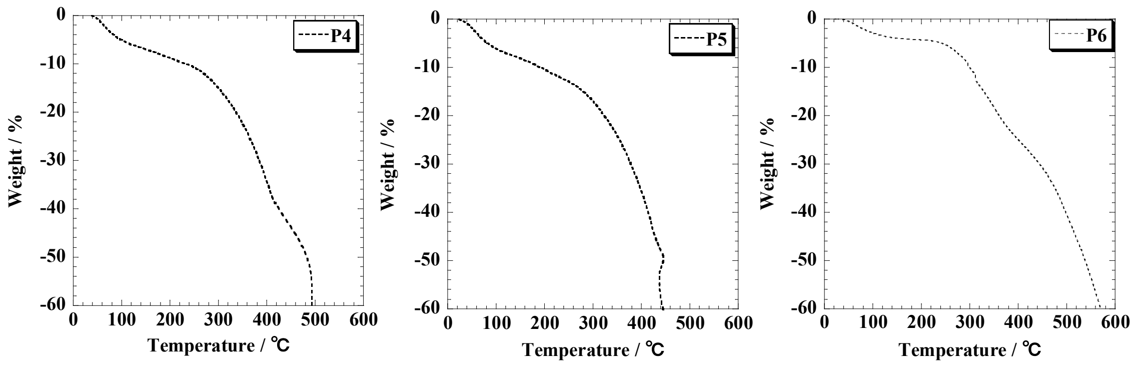

Figure S6. TGA curves of **P4-6** with a heating rate of 5 ºC / min in air.

Before measuring thermal gravimetric analysis (TGA), the polymers were dried under vacuum overnight.
